# Central Hyperthyroidism due to Thyroid-Stimulating Hormone-Secreting Pituitary Microadenoma in an Adolescent Boy: Case Report and Review of the Literature

**DOI:** 10.1155/2021/5573231

**Published:** 2021-03-20

**Authors:** Le Hoang Bao, Nguyen Minh Duc, Phan Cong Chien, Thieu-Thi Tra My, Tran Viet Thang, Tran Quang Nam

**Affiliations:** ^1^Department of Endocrinology, University Medical Center, Ho Chi Minh City, Vietnam; ^2^Department of Radiology, Hanoi Medical University, Ha Noi, Vietnam; ^3^Department of Radiology, Pham Ngoc Thach University of Medicine, Ho Chi Minh City, Vietnam; ^4^Department of Radiology, Children's Hospital 2, Ho Chi Minh City, Vietnam; ^5^Department of Radiology, University Medical Center, Ho Chi Minh City, Vietnam

## Abstract

Thyroid-stimulating hormone- (TSH-) secreting pituitary adenoma (TSH-oma) is a rare cause of secondary hyperthyroidism and can be misdiagnosed as primary hyperthyroidism. We report a case of a 15-year-old male patient who was one of two monozygotic twins and exhibited hyperthyroidism syndrome. The laboratory results showed secondary hyperthyroidism, with increased levels of free T3 (FT3) and free T4 (FT4) and no TSH inhibition. Magnetic resonance imaging (MRI) and histopathological examination of the pituitary gland confirmed pituitary microadenoma. The patient was treated with methimazole, propranolol, and somatostatin analogs to restore euthyroidism before undergoing an endoscopic transsphenoidal resection of the pituitary tumor. After surgery, the hyperthyroidism symptoms improved, thyroid hormones normalized, and MRI of the pituitary gland showed the complete removal of the tumor with no recurrence after 2 years of follow-up.

## 1. Introduction

Thyroid-stimulating hormone- (TSH-) secreting pituitary adenoma (TSH-oma) is a rare cause of hyperthyroidism [1], accounting for 0.5% to 3% of all functioning pituitary tumors and fewer than 1% of all hyperthyroidism cases [2]. The prevalence of TSH-oma in the general population is estimated to be 1-2 cases per million population [3]. TSH-omas are characterized by autonomous TSH secretion that is unresponsive to the negative feedback exerted by thyroid hormones [3]. This continuous TSH overstimulation leads to T4 and T3 hypersecretion, which is classified as either central hyperthyroidism or secondary hyperthyroidism [4]. Although Graves' disease and other common causes of hyperthyroidism typically occur more frequently in women than in men, TSH-omas occur with equal frequency in men and women, and the mean age at presentation ranges from 41 to 45 years [5, 6].

In the past, TSH-omas were diagnosed when they reached the invasive macroadenoma stage. Currently, unsuppressed TSH secretion in hyperthyroid patients is more readily detected by ultrasensitive immunometric assays for TSH and the measurement of circulating free thyroid hormones (FT4 and FT3) [3]. The current screening strategies that measure FT4 only when abnormal TSH levels are detected fail to recognize both central hypothyroidism and hyperthyroidism, leading to the misdiagnosis of TSH-oma [3]. In Vietnam, TSH-oma is a rare cause of hyperthyroidism and receives less attention than primary hyperthyroidism, and case reports remain limited.

## 2. Case Presentation

**Figure 1:**
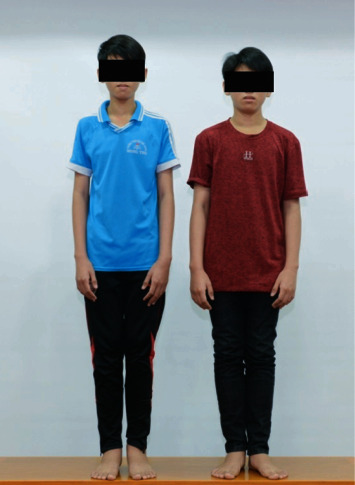
The patient (left) did not gain weight as well as his monozygotic twin brother (right).

A 15-year-old male patient presented to the clinic reporting exertion palpitations and feeling hot, with no other complaints. The patient had no growth chart until he came to our hospital. The patient did not gain weight as well as his monozygotic twin brother (Figure 1).

A physical examination revealed the following: pulse, 120 beats per minute; blood pressure, 140/80 mmHg; weight, 50 kg; height, 173 cm; body mass index (BMI) 16.7 kg/m^2^; and BMI percentile according to age, 6.1. No abnormalities in the heart, lung, abdomen, or nerve were detected. However, he presented signs of hand tremor at rest, and an enlarged thyroid gland was detected with a firm density. No eye abnormalities due to hyperthyroidism and no signs of acromegaly, gynecomastia, or galactorrhea were detected. He had normal secondary sexual characteristics, with his genitalia and pubic hair (Tanner stage 3). The patient had no history of prior thyroid disease. The monozygotic twin brother presented no clinical symptoms of hyperthyroidism, with a normal thyroid function test.

### 2.1. Investigation

The test results revealed secondary hyperthyroidism, with elevated FT3 levels at 23.08 pmol/L, FT4 levels of 86.32 pmol/L, and TSH levels of 8.25 mUI/L. Investigations of other hormones, including cortisol, adrenocorticotropic hormone (ACTH), testosterone, luteinizing hormone (LH), insulin-like growth factor-1 (IGF-1), and prolactin, showed normal results (Table 1).

**Figure 2:**
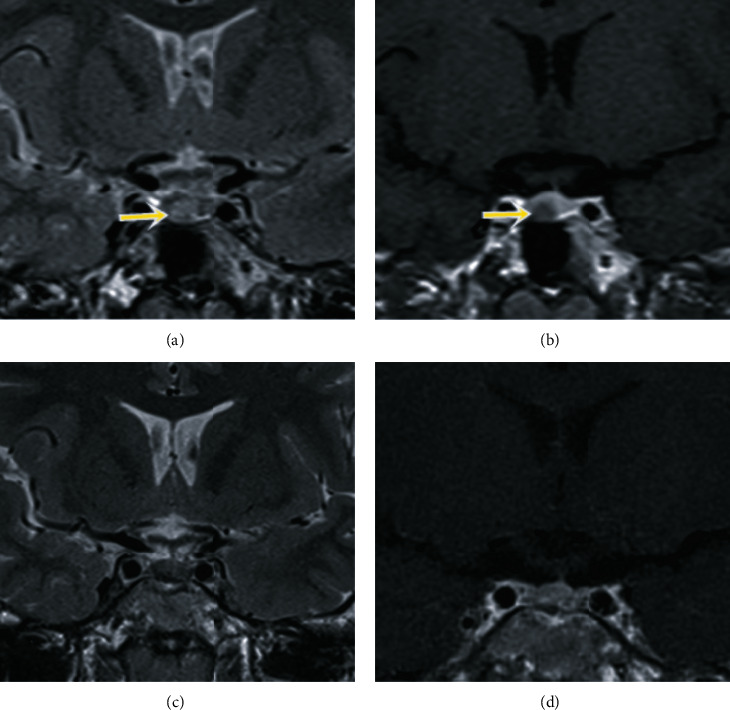
Preoperative (a, b) and postoperative (c, d) pituitary MRI results. Coronal T2-weighted image (a) and T1-weighted image with contrast enhancement (b) showed a pituitary lesion that was hyperintense on T2-weighted image and hypoenhancing compared with the surrounding pituitary parenchyma on T1-weighted image (arrows). The postoperative pituitary MRI showed no evidence of recurrence (c, d).

Thyroid ultrasound revealed that the thyroid gland was heterogeneous, without increased vascularity. Magnetic resonance imaging (MRI) of the pituitary showed a pituitary lesion with mild hyperintensity on the T2-weighted image (Figure 2(a)), which was less enhancing than the surrounding pituitary parenchyma (Figure 2(b)) and measured 5 × 6 × 7 mm in size. This lesion was suspected to be a pituitary microadenoma.

### 2.2. Treatment

**Figure 3:**
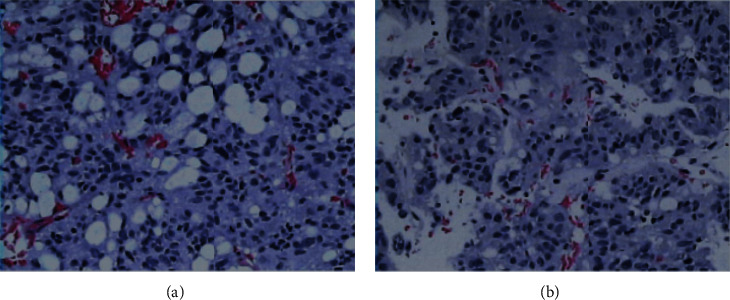
Hematoxylin and eosin staining of the patient's pituitary adenoma (a, b, × 200). Tumor tissue was composed of cytoplasmic-rich glandular cells arranged in clusters and featuring rounded nuclei. Cells arranged around the blood vessels and formed papillary structures with little mitosis.

The patient could not afford the cost of somatostatin analog; therefore, thyroid hormone levels were normalized by methimazole (20 mg per day) and propranolol (10 mg three times per day) before performing an endoscopic transsphenoidal resection of the pituitary tumor. When the patient was admitted to the hospital for surgery, he received a short-term treatment with sandostatin (0.1 mg three times a day), propylthiouracil (PTU), and propranolol. Because the patient was treated with oral methimazole 20 mg per day for 5 months and still had high serum FT4 before surgery, we switched to PTU to prevent thyroid storm during surgery. Postoperative pathology results revealed an adenoma of the pituitary gland (Figure 3).

**Figure 4:**
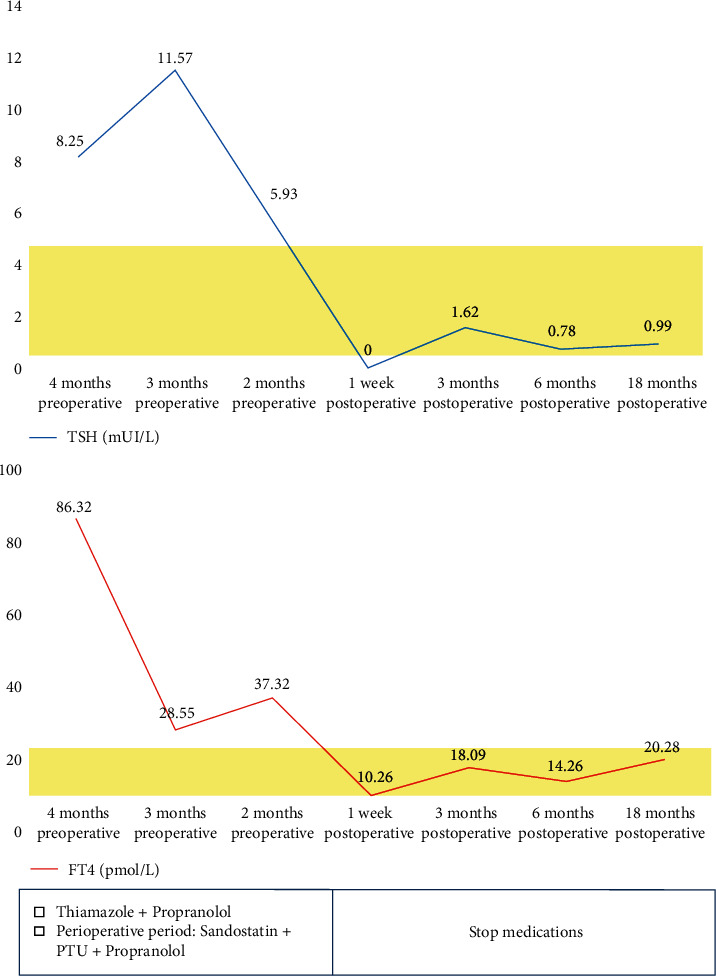
TSH and FT4 assay results showed decreasing trends after treatment.

After surgery, the doses of PTU and propranolol were gradually reduced. Two weeks following the operation, the patient was stabilized, PTU and propranolol were discontinued, and he was discharged from the hospital. Serum TSH and FT4 levels were evaluated and showed a decreasing tendency after treatment (Figure 4).

### 2.3. Outcome and Follow-Up

After discharge, the patient presented no clinical symptoms of hyperthyroidism. His weight was 50 kg before surgery and, 2 years later, it was 60 kg (gain 10 kg in 2 years); his height was 173 cm before surgery and later, his height was 175 cm. Hormonal testing after discharge was performed after 2 months, 6 months, and 18 months, and serum TSH, FT4, ACTH, and cortisol levels were detected in the normal range. MRI of the pituitary gland after 1 (Figure 2(c)) and 2 years (Figure 2(d)) showed the complete removal of the tumor, with no recurrence.

## 3. Discussion

We reported a case of a TSH-secreting pituitary adenoma that occurred in one of two monozygotic twins, who presented with exertion palpitations, feeling hot, trembling, lack of weight gain, tachycardia, and a diffuse, enlarged thyroid. With normal prolactin, IGF-1, cortisol, ACTH, testosterone, and LH levels, our patient was diagnosed with solitary TSH-oma, with no associated pituitary insufficiency. In 70% of cases, TSH-omas secrete TSH alone, and the remaining 30% are mixed adenomas that may cosecrete TSH and other anterior pituitary hormones (growth hormone, PRL, and LH/FSH; Table 2). The presence of these mixed adenomas can be explained by the expression of common transcription factors, such as Prop-1 and Pit-1, by thyrotrope, somatotrope, and lactotrophic cells [7].

Most patients have the typical symptoms and signs of hyperthyroidism (e.g., palpitations, tremors, and heat intolerance), but a few patients have mild or even no hyperthyroid symptoms [8]. In addition, patients may have symptoms related to the expanding tumor mass, such as the compression of the normal pituitary gland or the optic chiasm, or the secretion of growth hormone or prolactin [9]. The characteristic signs of Graves' ophthalmopathy (proptosis and periorbital edema) are absent without the coexistence of Graves' disease [10, 11].

The size of our patient's pituitary tumor on MRI was quite small, only 5 × 6 × 7 mm. In the past, MRI of the pituitary gland was more likely to detect a macroadenoma than a microadenoma [8], with an average tumor diameter of 3.1 cm [12]. In the last decade, microadenomas (diameter ≤1 cm) have been increasingly recorded (up to 30%–35%) due to the introduction of more sensitive and specific assays for the evaluation of thyroid function and increased awareness among endocrinologists and general practitioners [13].

Pediatric functioning pituitary adenoma is very rarely encountered in clinical practice; therefore, few pediatric cases of TSH-oma have been published in the literature (Table 3).

The diagnosis of TSH-secreting pituitary adenoma is critical to the success of treatment. According to Beck-Peccoz, approximately one-third of patients with TSH-oma underwent an inappropriate thyroidectomy or received radioiodine treatment due to an incorrect diagnosis of primary hyperthyroidism (i.e., Graves' disease or toxic multinodular goiter) [3].

The most appropriate definitive therapy for patients with TSH-omas is initial medical therapy to restore euthyroidism prior to a transsphenoidal resection of the tumor [2]. The preferred medical therapy is somatostatin analogs, which can reduce the size of the tumor and normalize serum thyroid hormone levels prior to surgery [3]. In a review of 43 TSH-secreting pituitary adenoma cases, 26 patients received somatostatin analogs as initial therapy, a reduction in TSH levels of greater than 50% occurred in 88% of cases, and the normalization of free T4 was reported in 85% of patients [8]. Somatostatin analogs are very expensive, and side effects include nausea, abdominal discomfort, bloating, diarrhea, glucose intolerance, and cholelithiasis. If somatostatin analogs are not well tolerated, dopamine agonists (such as bromocriptine and cabergoline) may be effective in select cases, particularly in patients whose tumors also secrete prolactin [8].

Antithyroid drugs (such as methimazole or PTU) can be combined with somatostatin analogs and propranolol to restore euthyroidism before surgery, according to the recommendations of the European Thyroid Association [2]. However, because our patient could not afford to pay for a somatostatin analog, we choose to use antithyroid drugs combined with propranolol to restore euthyroidism before the endoscopic transsphenoidal resection of the pituitary tumor [20].

The transsphenoidal resection of the pituitary adenoma is the definitive therapy of choice for patients with TSH-secreting adenomas. Adenoma resection cures the majority of patients with microadenomas and approximately 50%–60% of patients with macroadenoma [2, 8, 21]. Patients who do not recover following surgery can continue treatment with either radiotherapy or long-term somatostatin analogs [8].

The recommendations established by the European Thyroid Association in 2013 [2] confirmed the inability to clearly define cure criteria for TSH-oma patients after surgery or radiation therapy. The recommendations also outlined additional factors that could be considered when determining whether a cure has been achieved, including clinical improvements in hyperthyroidism and neurological symptoms and the absence of hormone and imaging abnormalities. Thus, our patient meets the posttreatment cure criteria, including no longer presenting symptoms of hyperthyroidism, the normalization of thyroid hormones, and the absence of a residual tumor on MRI.

Although no published protocols exist for TSH-omas, patients should be assessed clinically and biochemically 2 to 3 times during the first postoperative year and once per year thereafter. The evaluation should include the levels of TSH, FT3, FT4, and other pituitary hormones, as necessary. An MRI of the pituitary gland should be repeated every 2-3 years and should be performed if an increase in TSH and thyroid hormones is detected or clinical symptoms appear that suggest the potential for relapse [2].

Our patient was followed for 2 years after surgery and showed good results, with a normal thyroid function test and no evidence of recurrent tumor on pituitary gland MRI. In a series of 43 cases, which were followed for a mean of 8 years, two patients died (ages 75 and 86 years); 19 patients were cured after pituitary surgery alone; 17 patients displayed symptom of residual disease after surgery, which was controlled with somatostatin analogs, pituitary radiation, or both; and seven were controlled by somatostatin analogs alone [8].

## 4. Conclusion

TSH-oma is an uncommon cause of secondary hyperthyroidism, which can be easily misdiagnosed as primary hyperthyroidism. Other pituitary hormones should be evaluated to determine the presence of cosecretory tumors or pituitary insufficiency due to tumor compression. The principal treatment is initial medical therapy to normalize thyroid hormone levels prior to an endoscopic transsphenoidal resection of the pituitary tumor. This patient was cured due to an accurate preoperative diagnosis and multimodal therapy.

## Figures and Tables

**Table 1:** Hormonal results.

Investigations	Level	Reference range
FT4 (pmol/L)	**86.32**	12–22
FT3 (pmol/L)	**23.08**	3.1–6.8
TSH (mUI/L)	**8.25**	0.27–4.2
Cortisol (nmol/L)	261.3	118.6–618
ACTH (pmol/L)	9.2	1.6–13.9
Testosterone (nmol/L)	12.94	12–29
LH (mUI/mL)	2.55	1.24–8.62
IGF-1 (ng/mL)	291	100–1000
Prolactin (ng/mL)	16.64	4.04–15.2

FT4: free T4; FT3: free T3; TSH: thyroid-stimulating hormone; ACTH: adrenocorticotropic hormone; LH: luteinizing hormone; IGF-1: insulin-like growth factor-1. Bold levels are out of the normal range.

**Table 2:** Different types of TSH-secreting pituitary adenomas [3].

	No.	% of total
TSH-omas	461	100
Solitary TSH-oma	324	70.3
Mixed TSH-oma	137	29.7
TSH/GH-omas	84	18.3
TSH/prolactin-omas	45	9.7
TSH/gonadotropin-omas	8	1.7

TSH: thyroid secreting hormone; GH: growth hormone.

**Table 3:** Summary of the 7 cases reported in the literature of pediatric TSH-secreting pituitary adenomas and the present case.

Presentation	Tumor size	Function	Complications	Remission through surgery	Reference
15-year-old boy with hyperthyroidism	30 × 30 × 30 mm	TSH/GH/FSH/LH-omas	Inferior and superior extension	No	[14]
11-year-old girl after adenomectomy	48 × 62 × 58 mm	TSH/FSH-omas	Suprasellar, right temporal lobe extension	No	[15]
13-year-old boy with hyperthyroidism	20 × 15 mm	Solitary TSH-oma	Suprasellar extension Intratumor hemorrhage Diabetes insipidus Hypopituitarism Postoperative bacterial meningitis	No	[16]
16-year-old boy with goiter and hypertension	17 × 15 mm	TSH/FSH-omas	Suprasellar extension	No	[17]
13-year-old girl asymptomatic	28 × 25 × 29 mm	Solitary TSH-oma	Intrasellar and suprasellar extension	Successful treatment with somatostatin analogs	[18]
8-year-old boy with hyperthyroidism	Macroadenoma	Solitary TSH-oma	Suprasellar and sphenoidal extension	Yes	[19]
13-year-old boy with hyperthyroidism	40 × 45 mm	TSH/GH-omas	Compressing the surrounding structures Secondary adrenal insufficiency	No	[7]
15-year-old boy with hyperthyroidism	5 × 6 × 7 mm	Solitary TSH-oma	No	Yes	Present case

## Data Availability

Data sharing is not applicable to this article as no datasets were generated or analysed during the current study.
